# Phenome-Wide Scan Finds Potential Orofacial Risk Markers for Cancer

**DOI:** 10.1038/s41598-020-61654-3

**Published:** 2020-03-17

**Authors:** Mariana Bezamat, Benjamin Harrison, Yuqiao Zhou, Katherine M. Glickman, Vicente Telles, Christopher Guirguis, Adriana Modesto, Alexandre R. Vieira

**Affiliations:** 1 Department of Oral Biology, University of Pittsburgh, Pittsburgh, PA USA; 2 Department of Human Genetics, University of Pittsburgh, Pittsburgh, PA USA; 3 Department of Pediatric Dentistry, University of Pittsburgh, Pittsburgh, PA USA

**Keywords:** Cancer, Genetics, Biomarkers, Dental diseases

## Abstract

Cancer is a disease caused by a process that drives the transformation of normal cells into malignant cells. The late diagnosis of cancer has a negative impact on the health care system due to high treatment cost and decreased chances of favorable prognosis. Here, we aimed to identify orofacial conditions that can serve as potential risk markers for cancers by performing a phenome-wide scan (PheWAS). From a pool of 6,100 individuals, both genetic and epidemiological data of 1,671 individuals were selected: 350 because they were previously diagnosed with cancer and 1,321 to match to those individuals that had cancer, based on age, sex, and ethnicity serving as a comparison group. Results of this study showed that when analyzing the individuals affected by cancer separately, tooth loss/edentulism is associated with SNPs in *AXIN2* (rs11867417 p = 0.02 and rs2240308 p = 0.02), and leukoplakia of oral mucosa is associated with both *AXIN2* (rs2240308 p = 0.03) and *RHEB* (rs2374261 p = 0.03). These phenotypes did not show the same trends in patients that were not diagnosed with cancer, allowing for the conclusion that these phenotypes are unique to cases with higher cancer risk.

## Introduction

Cancer is a complex disease based on a process that drives the transformation of normal cells into their malignant derivatives^1^. Approximately 38.4% of people will be diagnosed with some form of cancer in their lifetime^2^. According to the American Cancer Society estimate for 2018, 1,735,350 people would be diagnosed with cancer in the United States, and an estimated 609,640 people would die of cancer^3^. However, survival rates vary depending on the type of cancer and the stage at diagnosis. Earlier stage diagnosis increases the likelihood of successful treatment and survival rates^2^. Nevertheless, because early cancers can be difficult to detect, much focus has been placed on the identification of more easily detectable cancer *risk markers*. A risk marker is a physiologic or anatomical characteristic that indicates a genetic tendency of developing the disease in question. The *BRCA1* and *BRCA2* genes are examples of genetic risk markers whose pathogenic variants can cause a significant increased risk of breast and ovarian cancer^4^. Additionally, several phenotypic risk markers (i.e., clinical traits), such as anatomical craniofacial abnormalities, have also been associated with an increased likelihood of developing cancers^5–12^. For example, cleft lip/palate has been found to be associated with diffuse gastric cancer^5^, breast cancer^6^, squamous cell carcinoma of the skin and others^7^. Additional studies reported associations between periodontitis and colorectal cancer^8^, breast cancer^9^ or any type of cancer^10^. Lastly, hypodontia (a disorder of tooth development) has been correlated with risk for epithelial ovarian cancer^11^ and colorectal cancer^12^. These previous reports point towards the hypothesis that orofacial phenotypes are related to cancer risk independent to cancer type. In other words, we can predict risk for cancer through the presence of genetic variants or through visual anatomical characteristics/ phenotypic traits that have been linked to an increased risk of developing the disease.

As a routine visit to the dentist includes examination of the oral cavity and head and neck structures, these professionals are uniquely positioned to identify these previously cited characteristics and conditions. A better understanding of a set of orofacial phenotypes that are markers for cancer risk could enable dentists and allied health care professionals to identify high risk patients. Furthermore, greater awareness of oral conditions that are linked to genetic predictors of cancer susceptibility will provide dentists an opportunity to improve patient outcomes by suggesting screenings for prevention.

There are two main methodologies used to detect gene-disease associations: an approach testing gene variants that can associate to one phenotype [targeted or genome-wide association scans (GWAS)] and an approach testing multiple phenotypes that can associate to one genetic variant (phenome-wide association scans or PheWAS). GWAS has proven to be an efficient method of identifying associations between gene variants, including single nucleotide polymorphisms (SNPs) and specific diseases. PheWAS is essentially “reverse GWAS” whereby one can determine the range of clinical traits (phenotypes) associated with a given genotype^13^. Results from several studies suggest that PheWAS can be used successfully to identify multiple associations from well-powered samples^13–16^ and provide novel insights not readily attainable by forward-genetic strategies. A unique quality of the PheWAS technique is its capacity to evaluate cross-phenotype associations or pleiotropy^17^. Pleiotropy is when one gene appears to affect more than one unrelated phenotypic trait. Oral phenotypes, especially, can have pleiotropic effects, such as in periapical pathology and periodontitis, which we identified in our previous study^18^. Identifying more of these effects and phenotypes, could provide us with a better understanding of drug development and how certain medications could act in different conditions.

In this present study, our hypothesis was that identifying different phenotypes associated with specific polymorphisms that may also be associated with cancer would allow us to determine which patients are at higher risk for this condition. To the best of our knowledge, this was the first time a PheWAS was applied to oral health outcomes, to identify clinical cancer risk markers.

## Results

We performed a phenotype-to-phenotype analysis, in which we compared the frequency of the most common orofacial conditions between cancer diagnosed individuals and a group of patients that were not diagnosed with cancer. As expected, the frequency of some oral diseases are high in the individuals participating in the Dental Registry and DNA Repository project. For example, among the 350 patients who reported having cancer, 84 have been diagnosed with periodontitis and 134 have been diagnosed with diseases of pulp and periapical tissues, versus 304 and 490 individuals out of 1,321 in the group without cancer for the same respective treatments. The most frequent condition was tooth loss/edentulism with 327 individuals being affected in the cancer diagnosed group versus 1147 in the group without cancer. We used these frequencies to calculate power, considering the incidence of tooth loss/edentulism in the affected group as 93%, and in the unaffected as 87%. Our total sample of 1,671 individuals gives 91% power to detect associations with an alpha of 0.05. When less frequent phenotypes or more similar incidence percentages within comparison groups are considered, the power decreases substantially. All additional power calculations for each individual condition are represented in Table 1.

**Table 1:** Oral conditions present in patients diagnosed with and without cancer and chi-square results.

Phenotype	Diagnosed with cancer (N = 350)	Non-diagnosed with cancer (N = 1,321)	P-value	Odds Ratio	Statistical Power
Diseases of pulp and periapical tissues	134	490	0.68	1.05	5.4%
Periodontitis (acute or chronic)	84	304	0.69	1.05	6%
Tooth loss/edentulism	327	1,147	**0.0006**	2.15	91%
Dental caries	237	843	0.17	1.18	28.2%
Anomalies of jaw size/symmetry	5	22	0.75	0.85	3.9%

The results showed that having tooth loss makes one more likely to have been diagnosed with cancer [327 out of 350 have tooth loss in the affected group and 1,147 out of 1,321 in the unaffected group (p = 0.0006, OR = 2.15, 95% C.I. 1.37–3.38)]. All the remaining phenotypes tested did not show any statistical difference between the two compared groups **(**Table 1**)**.

In the PheWAS analysis the criteria we used to select SNPs was based on our preliminary data results as well as results from previous studies performed by us and others^12,19–26^. Variation marking *AXIN2* (rs2240308 and rs11867417) have been shown to be associated with cancer and orofacial phenotypes such as cleft lip and palate and tooth agenesis^12,19–26^. Furthemore, the SNPs rs196929 (*ERN1*), rs2374261 (*RHEB*), rs1109089 (*RHEB*), rs4396582 (*RAPTOR*) showed association with three oral phenotypes (dental caries, periodontitis, and periapical lesions) in our previous study from a different Dental Registry and DNA Repository cohort^18^. Those SNPs are present in pathways involved in cell proliferation, differentiation and inflammation, and may contribute to cancer risk as well.

**Figure 1:**
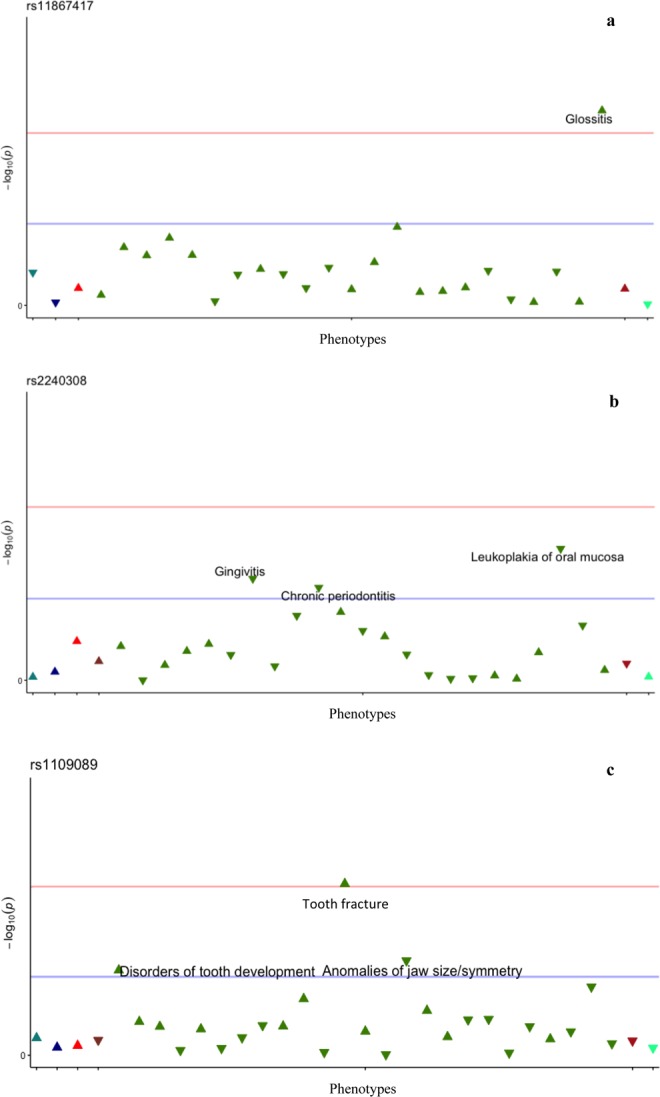
Plot representing the phenome-wide association analysis in the total sample. The horizontal red line indicates the threshold of p = 0.002; the horizontal blue line indicates the threshold of p = 0.05, phenotypes found below the blue line (p > 0.05 – not associated) are not annotated in the plots to avoid noise. The triangle tip direction represents the odds ratio direction of each association, upward triangles indicate OR ≥ 1; downward triangles indicate a protective effect (OR < 1.0); different triangle colors indicate different disease groups (from left to right – dark green = neoplasms, dark blue = neurological system, bright red = circulatory system, brown = respiratory, green = digestive, dark red = dermatologic and light blue = congenital anomalies). **(a)** *AXIN2* - rs11867417 and its association with glossitis (p < 0.002). **(b)** *AXIN2* - rs2240308 and its protective effect towards having gingivitis, chronic periodontitis, and leukoplakia of the oral mucosa (p < 0.05). **(c)** *RHEB* - rs1109089 and its association with both disorders of tooth development (p < 0.05) (p < 0.05), and tooth fracture (p < 0.002) (p < 0.002), and its protective effect towards anomalies of jaw size/ symmetry (p < 0.05).

**Figure 2:**
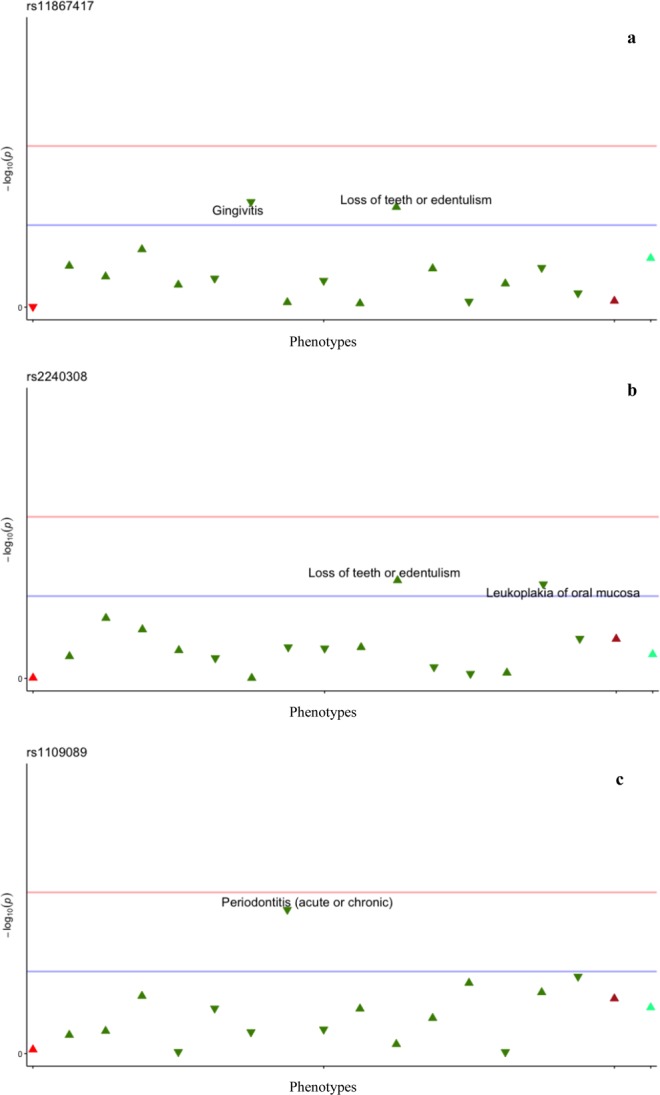
Plot representing the phenome-wide association analysis in the cancer-affected sample. The horizontal red line indicates the threshold of p = 0.002; the horizontal blue line indicates the threshold of p = 0.05, phenotypes found below the blue line (p > 0.05 – not associated) are not annotated in the plots to avoid noise. The triangle tip direction represents the odds ratio direction of each association, upward triangles indicate OR ≥ 1; downward triangles indicate a protective effect (OR < 1.0); different triangle colors indicate different disease groups (from left to right – light red=circulatory system, green=digestive, dark red=dermatologic and light blue=congenital anomalies). **(a)** *AXIN2* - rs11867417 and its association with loss of teeth/edentulism, and its protective effect towards gingivitis (p < 0.05). **(b)** *AXIN2* - rs2240308 and its association with loss of teeth/edentulism and its protective effect against leukoplakia of the oral mucosa (p < 0.05). **(c)** *RHEB* - rs1109089 and its protective effect against periodontitis (p < 0.05).

The PheWAS analysis **(**Table 2**)** revealed several suggestive associations between craniofacial phenotypes and the SNPs tested. However, there were no significant associations after Bonferroni correction. A trend for association for association was found between *AXIN2* rs11867417 minor allele and the presence of glossitis (p = 7.80E-04, OR = 2.48, 95% C.I. 1.49–4.36). Figure 1 illustrates the most substantial results in the total sample. We set a threshold value of p = 0.002 (horizontal red line) in all Manhattan plots in order to facilitate visualization of trends for association. The horizontal blue line represents the p = 0.05 threshold, phenotypes found below the blue line are not annotated in the plots to avoid noise. The triangle tip direction represents the odds ratio direction of each association. In order to identify whether these associations were preferentially linked to the individuals with a cancer condition in our population, we ran PheWAS in both cancer-affected **(**Fig. 2**)** and unaffected samples separately. Table 3 shows the results obtained in the cancer-affected sample and Table 4 shows the results obtained after analysis of the cancer-unaffected sample. When analyzing the cancer affected group separately, tooth loss/edentulism and leukoplakia of oral mucosa are within the phenotypes that showed trends for association with a number of different SNPs. Interestingly, when the comparison group was analyzed, no significant associations with these phenotypes were identified, leading us to suggest that they are possibly unique to the cancer affected sample.

**Table 2:** PheWAS results in the total sample.

Phecode	Description	SNP/ Allele	lower	upper	Odds Ratio	P value	Affected by the disease described	Non-affected by the disease described	Allele frequency
529.1	Glossitis	rs11867417_C	1.498	4.368	2.486	7.80E-04	39	1088	0.59405501
525	Tooth fracture	rs2374261_T	1.187	1.849	1.480	5.08E-04	181	1289	0.436735
		rs1109089_T	1.146	1.772	1.424	1.43E-03	186	1356	0.44131
528.6	Leukoplakia of oral mucosa	rs2240308_A	0.676	0.942	0.799	7.97E-03	391	983	0.4152111
526.4	Temporomandibular joint disorder	rs2374261_T	1.045	1.409	1.213	1.08E-02	555	915	0.4367347
523.1	Gingivitis	rs2240308_A	0.703	0.974	0.828	2.39E-02	406	968	0.4152111
526.3	Anomalies of jaw size/symmetry	rs1109089_T	0.207	0.880	0.446	2.69E-02	20	1522	0.44131
523.32	Chronic periodontitis	rs2240308_A	0.645	0.980	0.796	3.33E-02	211	1163	0.4152111
520	Disorders of tooth development	rs1109089_T	1.021	2.149	1.477	3.89E-02	58	1484	0.44131
528.11	Stomatitis and mucositis	rs4396582_G	1.015	1.949	1.403	4.07E-02	78	1427	0.4744186

Logistic regression using the additive genomic model was performed and the table shows the nominal results (p values between 0.00025 and 0.05). Significant results were not identified.

**Table 3:** PheWAS results in the patients that had cancer.

Phecode	Description	SNP/ Allele	lower	upper	Odds Ratio	P value	Affected by the disease described	Non-affected by the disease described	Allele frequency
523.3	Periodontitis (acute or chronic)	rs1109089_T	0.384	0.841	0.572	0.0052	80	240	0.44375
		rs2374261_T	0.383	0.858	0.578	0.0076	75	239	0.433121
523.1	Gingivitis	rs11867417_C	0.413	0.927	0.623	0.0213	70	169	0.5669456
		rs4396582_G	0.483	0.971	0.688	0.0355	106	220	0.4631902
529	Diseases of the tongue	rs2374261_T	0.489	0.951	0.685	0.0258	142	172	0.433121
525.1	Tooth loss/edentulism	rs2240308_A	1.134	4.979	2.269	0.0281	263	22	0.4087719
		rs11867417_C	1.108	4.016	2.066	0.0258	217	22	0.5669456
528.6	Leukoplakia of oral mucosa	rs2240308_A	0.448	0.945	0.655	0.0323	86	199	0.4087719
		rs2374261_T	1.029	2.096	1.464	0.0348	95	219	0.433121

Logistic regression using the additive genomic model was performed and the table shows the nominal results (p values between 0.00025 and 0.05). Significant results were not identified.

**Table 4:** PheWAS results in the patients that did not have cancer.

Phecode	Description	SNP/ Allele	lower	upper	Odds Ratio	P value	Affected by the disease described	Non-affected by the disease described	Allele frequency
525	Tooth fracture	rs2374261_T	1.140	1.884	1.464	2.89E-03	137	1019	0.4377163
		rs1109089_T	1.130	1.850	1.444	3.37E-03	141	1081	0.440671
		rs2240308_A	0.576	0.983	0.755	3.94E-02	122	967	0.4168962
523.32	Chronic Periodontitis	rs2374261_T	1.067	1.675	1.336	1.14E-02	178	978	0.4377163
		rs1109089_T	1.020	1.572	1.266	3.20E-02	193	1029	0.440671
529.1	Glossitis	rs11867417_C	1.211	3.914	2.113	1.16E-02	31	857	0.6013514
523.1	Gingivitis	rs2240308_A	0.659	0.949	0.792	1.21E-02	320	769	0.4168962

Logistic regression using the additive genomic model was performed and the table shows the nominal results (p values between 0.00025 and 0.05). Significant results were not identified.

## Discussion

Here we report an analysis of a cohort enriched with individuals diagnosed with cancer using PheWAS in an attempt to identify oral health outcomes and genetic variants that may be indicators of cancer risk. nominal associations were found when the cancer-affected patients were analyzed separately. For both SNPs in *RHEB*, the less frequent alleles appeared to be protective of having periodontitis in the cancer diagnosed individuals, and having anomalies of jaw size/ symmetry in the total sample. Both *RHEB* and *RAPTOR* genes are present in the signaling pathway known as the mammalian target of rapamycin (*mTOR*). The *mTOR* signaling is a master regulator of protein synthesis, *RHEB* (Ras homolog enriched in brain) is a positive regulator of *mTOR* and is located in the center of the signaling pathway^27^. *RAPTOR* (the Regulatory Associated Protein of mTOR) regulates cell growth in response to nutrient and insulin levels^28^. Activation of *mTOR* promotes tumor growth and metastasis^29^. *Raptor* knockout mice display facial growth deficiency, including mandible^30^, which is consistent with our finding.

Associations were also identified for a number of other markers such as between two markers in *RHEB* and leukoplakia of the oral mucosa and two markers in *AXIN2* and loss of teeth/edentulism and, both phenotypes unique to the cancer-affected group. *AXIN2* is a component of Wnt signaling and is expressed in the dental mesenchyme, dental papilla and enamel knot^31^. Our results confirm a previously suggested role of *AXIN2* in tooth agenesis^19,32^. No significant associations were found when analyzing the cancer-affected group in separate (after Bonferroni correction). This may be due to the reduced power of the smaller sample size of the cancer-affected group. Nevertheless, the p-values below 0.00025 set after Bonferroni correction may be too strict and lead to missing true biological signals^33^.

The phenotype-to-phenotype analysis showed an association between having had tooth loss and having been diagnosed with cancer, consistent with the results obtained in the PheWAS analysis. Since not only tooth loss/edentulism but also leukoplakia of oral mucosa are examples of phenotypes that showed associated in individuals diagnosed with cancers, different types of cancers could be better defined to confirm if these oral health outcomes associate. Similarly, when genetic variation was analysed as potential risk markers in the total sample, some of the results after correction for multiple testing suggest that the risk alleles are not overrepresented among individuals affected by cancer, making it difficult to use those specific phenotypes as markers of risk.

This is the first time that a phenome-wide study has been performed using a dental database and we demonstrated the applicability of the technique to the dental field and dental researchers for future studies. However, a few limitations were experienced. We were not able to differentiate between losing one tooth, including third molars, and losing all teeth (edentulism). Refining these and other phenotypes in future studies, is an approach that will help clarify if edentulism, which is an extreme outcome, is a risk marker for cancer. The second limitation we faced here is that the types of cancer present in our study sample are not representative of the most frequent cancers in the general population. Lung cancer, for example, is the second most common cancer, for both men and women. However, in our Dental Registry and DNA Repository project, only ten subjects (four males and six females) reported having lung cancer. The reason for this difference might be explained by the high mortality rate of lung cancer in patients. For a patient to participate in the Dental Registry and DNA Repository project and report having had cancer, they either survived the disease or are undergoing treatment. Therefore, there is a higher probability that these individuals had a type of cancer with a low five-year survival rate and were not captured in our sample. Further, ideally we would be able to replicate our work in another cohort, but our project is the only one in the world that includes over 40 specific oral phenotypes that were diagnosed by a careful dental exam. Dental phenotypes especially are typically omitted from such studies since they are not part of medical records.

Analyses were done taking into consideration sex and ethnicity. Females and males share a genome but differ in almost every phenotype^34^, including oral health outcomes such as dental caries^35^. We used self-reported ethnicity as an adjustment in the regression analysis, and we are aware that there are instances that some self-identified African Americans may have a high percentage of European ancestry, whereas some self-identified European Americans have substantial admixture from African ancestry^36^. To mitigate the potential effect of population substructure, ancestry may be derived from genetic data. Our previous experience with the data from the Dental Registry and DNA Repository project suggests that there is good consisitency between self-reported and genetically driven ethnicity definitions^37^. Comparisons between estimates of genetic ancestry and self-reported ethnicicty in African and European American populations from 1000 genomes project datasers showed that European ancestry estimations from genetic data was 97.6% for individuals that self-reported as Europeans, only 1.3% for individuals that self-reported as Africans, and 10.8% for individuals that self-reported as African Americans^36^. The analysis could also not account for known factors that modify oral health outcomes. We did not include a surrogate for socioeconomic status in the analysis, however the participants of our Dental Registry and DNA Repository project are for the most part, from lower socioeconomic status and have poor oral and overall health outcomes^38^.We also could not include a measure for the potential consequence of cancer on the patient’s oral health. Cancer treatment can be as devastating as the disease itself, with the aggravating factor that dentists can be perceived as less knowledgeable about cancer treatment-related oral concerns and therefore trusted less than oncologists^39,40^.

In summary, previously suggested associations in the studied genes were consistent with our findings and novel potential associations were identified. Tooth loss/edentulism was associated with two *AXIN2* SNPs in the cancer-affected sample, increasing up to 2.3 times the chances of losing teeth. The phenotype-to-phenotype analysis showed similar results, confirming that individuals diagnosed with cancer experience more tooth loss. This particular association could be just the result of the cancer itself, since most of the cancer diagnosed patients have immunosuppression, which consequently may lead to tooth loss. However, one should consider that a particular phenotype that is the result of a person’s cancer still may be more likely to be identified prior to the cancer itself being identified. Individuals with immune system disorders, such as Dubowitz or Down syndromes, show characteristic facies and dental abnormalities and higher incidence of leukemia/lymphoma^41^.

This study implemented a novel strategy to identify cancer risk markers by combining electronic health records and genetics. Identification of individuals carrying craniofacial and genetic markers allow dentists to refer them for screenings/checkups more frequently. This conduct potentially increases the possibility of preventing cancers or diagnosing them at early stages when the treatment survival rates are higher.

## Methods

**Figure 3:**
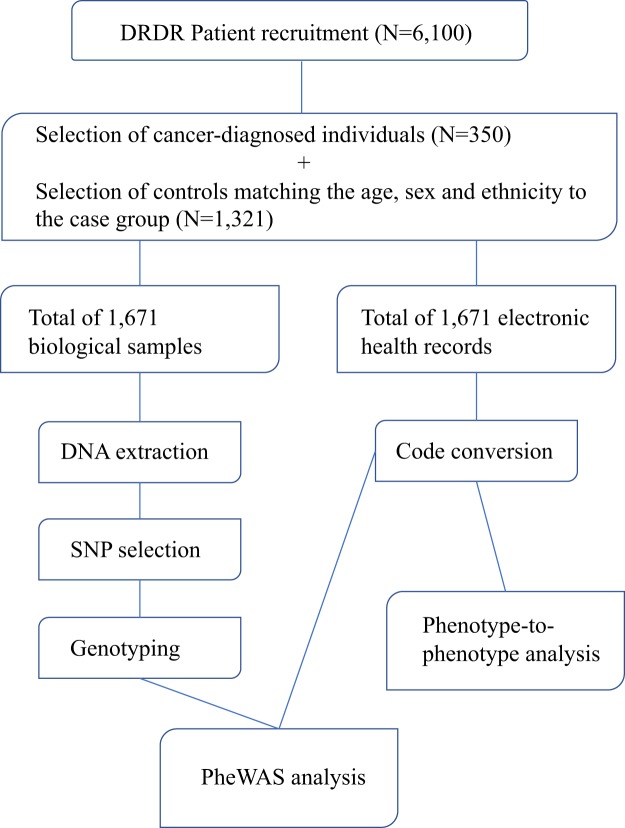
Overall study design.

Data from the Dental Registry and DNA Repository project available at the University of Pittsburgh was used. This project has the approval of the University of Pittsburgh Institutional Review Board (IRB # 0606091). All methods were performed in accordance with the guidelines and regulations. When data were collected, approximately 6,100 unrelated individuals who provided written informed consent were available for this project^38,42^. Biospecimens were linked to patients’ complete electronic health record (EHR) data (available on REDCap system), thus permitting analysis of associations between genetic variation obtained from DNA extracted from the specimens and dental and medical conditions. All data were deidentified, and biospecimens were linked to EHRs using a unique study number rather than personal identifying information. Complete medical and dental records, radiographs, oral photographs, and information about possible risk factors for cancer and other chronic conditions were available, under specific codes created for the project. From the study database, a total of 350 individuals who have been diagnosed with cancer were first selected for the study. Then, a comparison group comprised of individuals who have never received a cancer diagnosis and were matched to the 350 patients in the experimental group by age, ethnicity, and sex reaching a 1:4 ratio was selected. Table 5 shows the distribution of the study sample and Fig. 3 describes the overall study design.

**Table 5:** Study sample characteristics.

	Individuals with a Diagnosis of Cancer (n = 350)	Matched Individuals without a Diagnosis of Cancer (n = 1,321)
**Age in years (mean, range)**	60.9 (13–91)	60.6 (13–97)
**Sex (n, %)**		
Female	187 (53.43%)	719 (54.43%)
Male	163 (46.57%)	602 (45.57%)
**Self-reported Ethnicity (n, %)**		
White	265 (75.71%)	1,042 (78.88%)
Black	75 (21.43%)	266 (20.14%)
Asians	2 (0.57%)	7 (0.53%)
Hispanics	3 (0.86%)	6 (0.45%)
Other	5 (1.43%)	0 (0.00%)

The most common types of cancer in the study population are described by sex in Table 6. Phenotypes examined in this study included dental caries, diseases of the dental pulp and periapical tissues, dental abscess, diseases of the jaw, missing teeth or edentulism, acute periodontitis, chronic periodontitis, disorders of tooth development or eruption, tooth fracture, sleep related movement disorders (e.g., bruxism), diseases of salivary glands, malocclusion, stomatitis, mucositis, erythema, lingual varicose veins, diseases of the tongue, temporomandibular joint disorder, hemangioma, lymphadenitis, candidiasis, thyroid disorders, and lacrimal gland disorders.

**Table 6:** Most common cancers in the sample by sex.

Male		Female	
Cancer type	# Cases	Cancer type	# Cases
Prostate	38	Breast	56
Skin	35	Skin	29
Lymphoma	12	Cervix	19
Kidney	10	Colon/rectal	17
Rectal	9	Thyroid	12

### Phenotype-to-phenotype analysis

We matched individuals diagnosed with cancer with individuals without cancer according to their age, ethnicity and sex, since these variables associate with the onset or frequency of many outcomes we selected to study. Then, we used simple chi-square (alpha = 0.05) to ascertain if particular dental outcomes preferentially associated with each other. The frequency of the most common head and neck conditions in the group of individuals who received a diagnosis of cancer was compared with the group of individuals who were not diagnosed with cancer. We tested phenotypes such as the presence of diseases of pulp and periapical tissues, periodontitis (acute or chronic), tooth loss/edentulism, dental caries and anomalies of jaw size/symmetry.

### Genomic polymorphisms

We have selected SNPs based on our preliminary data where we tested 27 markers in eight genes of two pathways involved with cell proliferation and homeostasis^18^. As a result of our previous study, the SNPs rs196929 (*ERN1*), rs2374261 (*RHEB*), rs1109089 (*RHEB*), rs4396582 (*RAPTOR*) showed associations with three oral phenotypes (dental caries, periodontitis, and periapical lesions). Those SNPs are present in pathways involved in cell proliferation, differentiation and inflammation, and may contribute to cancer risk as well. We also tested variation marking *AXIN2* (rs2240308 and rs11867417), based on its association with cancer in different populations as well as craniofacial phenotypes such as cleft lip and palate and tooth agenesis, reported in previous studies^12,19–26^. Table 7 lists the genes, the selected SNPs and their minor allele frequencies (MAF).

**Table 7:** Selected SNPs.

Gene	SNP	MAF
*ERN1*	rs196929	T = 0.4046
*RHEB*	rs2374261	T = 0.3900
	rs1109089	T = 0.3958
*AXIN2*	rs2240308 rs11867417	A = 0.3377
		C = 0.4675
*RAPTOR*	rs4396582	G = 0.4113

### DNA extraction

Genomic DNA was extracted from salivary samples of the 1,671 individuals using established protocols^43^. In order to run the polymerase chain reaction (PCR) using the selected SNPs, DNA samples were diluted in Tris- EDTA (TE) buffer to a concentration of 2 ng/μl. Then, a volume of 1.0 μl was transferred to PCR plates and 2.0 μl of reaction mix containing master mix, water and the SNP of interest was added to each well of the 384 well plate. Reactions were carried out using Taqman chemistry in volumes of 3.0 μl in an ABI PRISM Sequence Detection System 7900, software version 1.7 (Applied Biosystems, Foster City, CA, USA). Genotypes were generated blindly to clinical diagnosis status. The feasibility of this methodology was established in our preliminary study where we identified the SNPs involved in oral phenotypes^18^.

### Code conversion

As the Dental Registry and DNA Repository project uses internal specific codes that better describe dental conditions instead of the more general International Classification of Diseases - Ninth Revision (ICD-9), and the PheWAS package in R studio only reads ICD-9 codes or “Phecodes”, we included as part of our strategic approach the conversion of our internal codes into “Phecodes” to be able to run the PheWAS. Treatments and phenotypes were recoded and identified by “Phecodes” and each tooth might have more than one code according to the number of different phenotypes in the tooth. The treatment provided is important to help us determine whether the tooth had previous dental decay, successive restorations’ failures or unsuccessful treatments leading to extractions for example. The way the program is written, the use of universal codes or “Phecodes” is required for the analytic software to perform the analysis of these data. The raw data was gathered from the Dental Registry and DNA Repository project through REDCap (Research Eletronic Data Capture) hosted at the University of Pittsburgh^44^. Data were exported in the form of an Excel file, which was converted to a Comma Separated Variable file (.CSV). The.CSV file was then read and processed by a script that converted all relevant codes from project’s internal form to their Phecode form. A program was written in Javascript to read the.CSV file. A list of valid conversions was manually created by us according to the codes we have available in our project and a phecode catalog map that can be found at www.phewascatalog.org - the codes can be identified by either typing the correspondent ICD9 code or the phenotype of interest. The list also in the.CSV form, was entered into the script, and the program replaced all occurrences of relevant raw codes to their Phecode form and a “true or false” file was manually created for each of the phenotypes in a particular individual. This final file was then uploaded into R to be used in the phewas analysis.

### PheWAS statistical methods and power calculation

The R software has a PheWAS package that generates perfect matches between affected individuals and their comparators for each individual set of phenotypes. Each phenotype includes an optional set of exclusion phenotypes for similar diagnoses to more accurately identify true controls. This step prevents patients with similar diseases from being marked as a control during the statistical analysis^45^. The current PheWAS map and PheWAS script written in R is available at http://phewascatalog.org^45^. The standard PheWAS statistical test is a logistic regression that calculates odds ratios, p-values, and includes Bonferroni correction to account for multiple testing. We used the additive genomic model, assuming that each allele contributes a fixed amount of risk that is additive. We incorporated sex and ethnicity as covariates in the logistic regression analysis in order to adjust for potential confounding effects.

According to a simulation study that investigated power estimates in PheWAS, a sample size of 200 cases or more achieves 80% statistical power to identify associations for common variants. In addition, a sample size of 1,000 or more individuals performed best in the simulations^46^. Our total sample consists of 1,671 individuals, 350 diagnosed with cancer and 1,321 non-affected by cancer, which gives an approximate 1:4 case-control ratio. Considering sample size, case-control ratio, and minor allele frequencies of our SNPs **(**Table 7**)**, the analysis of the cohort defined by having cancer will have a power of 100% to detect possible associations with α at 0.00025.

## Data Availability

The dataset generated and/or analysed during the current study are available from the corresponding author on reasonable request.
